# Identification of infection by Chikungunya, Zika, and Dengue in an area of the Peruvian coast. Molecular diagnosis and clinical characteristics

**DOI:** 10.1186/s13104-018-3290-0

**Published:** 2018-03-14

**Authors:** José Sánchez-Carbonel, Derek Tantaléan-Yépez, Miguel Angel Aguilar-Luis, Wilmer Silva-Caso, Pablo Weilg, Fernando Vásquez-Achaya, Luis Costa, Johanna Martins-Luna, Isabel Sandoval, Juana del Valle-Mendoza

**Affiliations:** 1 School of Medicine, Research and Innovation Centre of the Faculty of Health Sciences, Universidad Peruana de Ciencias Aplicadas, Lima, Peru; 2 Laboratorio de Biología Molecular, Instituto de Investigación Nutricional (IIN), Lima, Peru; 3 Instituto de Investigación de Enfermedades Infecciosas (IIEI), Lima, Peru; 4 Departamento de Ciencias Básicas, Escuela de Ciencias de la Salud, Universidad de Viña del Mar, Viña del Mar, Chile; 5 Red de Salud de Morropon Chulucanas, Dirección Regional de Salud de Piura (DIRESA-Piura), Piura, Peru

**Keywords:** Peru, Arbovirus, Dengue, Chikungunya, Zika, PCR

## Abstract

**Objective:**

To assess the presence of Dengue, Chikungunya, and Zika in serum samples of patients with acute febrile illness in Piura, Peru and describe the most common clinical features.

**Results:**

Dengue was the most common arbovirus detected in 170/496 (34.3%), followed by Zika in 39/496 (7.9%) and Chikungunya in 23/496 (4.6%). Among the 170 samples positive for Dengue, serotype 2 was the most predominant type present in 97/170 (57.1%) of samples, followed by the serotype 3 in 9/170 (5.3%). Headaches, muscle pain, and joint pain were the most common symptoms associated with fever in patients with Dengue and Zika. No symptoms predominance was observed in patients with Chikungunya.Dengue is considered the most frequent arbovirus in Peru and the number of cases has increased dramatically in the last 5 years. However, it is not the only arbovirus that circulates along the northern coast of Peru. It has also been determined the presence of Zika and Chikungunya in our population, which may suggest the circulation of other arboviruses that have not been detected.

## Introduction

In the last years, the incidence of emerging and reemerging arboviral diseases has increased producing a serious social and economic impact in Latin America [1–4]. Dengue (DENV), Chikungunya (CHIKV) and Zika (ZIKV) are arboviruses of great concern due to their impact on public health especially in low-income countries [3, 5, 6].

Dengue which is considered the predominant arboviral disease in Peru has experienced a rapid prevalence increase in the last 5 years. In 2017, the number of cases has tripled compared to the previous year being the Peruvian coasts the most affected areas [7]. Piura which is located on the Northwestern coast of Peru registered 26.2% of cases in 2016 and is by far the region with the higher prevalence of DENV, followed by La Libertad (17.2%), Ayacucho (12.1%) and Loreto (10.8%) [8, 9]. However, due to the limited laboratory confirmation rate and the number of asymptomatic infected patients, data available at the national level are still limited [10].

In 2013, the first native case of Chikungunya was reported on the Caribbean Island of San Martin [11]. Furthermore, CHIKV has also experienced a rapid spread and by the year 2016 more than 152,000 cases of Chikungunya fever have been reported in Latin America, with most cases reported in Bolivia, Ecuador, Colombia, Venezuela and Peru [12–14].

Zika was detected in Peru for the first time in 2016, and until now more than 865 confirmed cases have been registered [15, 16]. ZIKV have been reported in 10 of Peru’s 25 departments including Cajamarca, Ica, La Libertad, Madre de Dios, and Piura. However, the impact of Zika in the Peruvian communities has acquired special attention in the last year due to the potential effects on pregnancy. Since ZIKV was first reported in Peru, more than 200 confirmed cases of pregnant women affected by the virus have been observed [16].

The acute febrile illness which can be associated with nausea, vomiting, muscle pain, joint pain, rash, abdominal pain among others [3, 5, 6]. Therefore, laboratory confirmation has become an essential tool for the etiological diagnosis of arboviruses. In addition, molecular techniques such as PCR have proven to be the most sensitive diagnostic method for the detection and confounding of infections by DENV, ZIKV, and CHIKV [3, 17, 18].

In Peru, arboviral infections represent a major health concern [19]. Additionally, due to the limited laboratory resources etiologies such as Chikungunya or Zika are commonly misdiagnosed in dengue-endemic regions such as Piura, Peru. The present study main objective was to describe the prevalence of DENV, CHIKV, and ZIKV via real-time RT-PCR in patients with acute febrile illness from Piura, Peru.

## Main text

### Methods

#### Patients and sampling

We conducted a descriptive Cross-sectional study using convenience sampling. Data collection was performed by experienced personnel trained in the health establishments who were responsible for filling out epidemiological data sheets. The study was conducted in Piura in coordination with the *“Dirección Regional de Salud de Piura* – *Red de Salud Morropón* -*Chulucanas*”. Piura is a coastal region in Northwestern Peru which has been extensively recognized as an endemic area for dengue [8, 9, 19].

In this study, 496 patients with an acute febrile illness were evaluated, who met our inclusion criteria were studied from May to August 2016.

Inclusion criteria: Patients who presented to the to health establishment with acute febrile illness (greater than or equal to 38 °C axillary temperature for less than 7 days) along with one or more of the following symptoms: chills, headache, dizziness, cough, sore throat, nausea and/or vomits, loss of appetite, back pain, dysuria, myalgias, arthralgias, retro-ocular pain, rash, melena, nasal bleeding, gums bleeding, petechiae, ecchymosis, blood tinged sputum, abdominal pain, chest pain, fatigue and altered mental status.

#### Exclusion criteria

Patients with samples inadequately conserved, or with an incomplete data sheet.

#### Ethics statement

This study was approved by the Research Ethics Board of the *Hospital Regional de Cajamarca,* Peru. A written informed consent was signed before enrollment; for participants under 18 years old the informed consent was signed by parents or children caregivers before enrollment.

#### Samples

One serum sample per patient was collected by using Vacuette^®^ TUBE Serum Separator Clot Activator (Vacuette, Greiner Bio-One, Kremsmünster, Austria); all the samples were stored at − 80 °C.

The Centers for Disease Control and Prevention (CDC, Fort Collins, CO, USA) provided positive controls for DENV, CHIKV, and ZIKV.

#### RNA extraction

From 200 μL of serum samples, RNA was extracted with the High Pure RNA Isolation Kit (Roche Applied Science, Mannheim, Germany), according to the manufacturer’s instructions.

#### Amplification by Real-time RT-PCR assay for detection DENV, CHKV and ZIKV with TaqMan probe

A one-step RT-PCR was performed using TaqMan with BHQ quencher probe at 125 nM and 250 nM of primers in a final volume of 20 μL. Five microliters of the extracted RNA was combined with 15 µL of the master mix and the reverse transcription step was performed 95 °C for 15 min, 60 cycles of 15 s at 95 °C and 45 s at 60 °C. All the procedure was performed in Light Cycler^®^ 2.0 Instrument and data was analyzed with the LightCycler^®^ Software 4.1 (Roche Diagnostic, Deutschland-Mannheim, Germany). The primers and the probe used for DENV, CHIKV, and ZIKV described by Leparc-Goffart et al. [20], Peyrefitte et al. [21] and Faye et al. [22], respectively.

#### Data analysis

Qualitative variables were reported as frequencies and percentages. All analyses were processed with the IBM Statistical Package for the Social Sciences (SPSS) software version 21.0 (SPSS, Chicago, IL, USA).

### Results

A total of 496 serum samples from patients with acute febrile illness were assessed for the presence of DENV, CHIKV, and ZIKV. Most of the patients were between 20 and 44 years old (31.3%) and between 5 and 19 years old (27.8%) with no gender predominance. DENV was the most common arbovirus detected in 170/496 (34.3%), followed by ZIKV in 39/496 (7.9%) and CHIKV in 23/496 (4.6%) **(**Table 1).

**Table 1:** Demographics and PCR confirmed of DENV, ZIKV, and CHIKV in patients with acute febrile illness

Demographics	Totality of cases n = 496 (%)	PCR real time confirmed cases		
		DENV (n = 170)	ZIKV (n = 39)	CHIKV (n = 23)
		n (%)	n (%)	n (%)
Age (years)				
0–4	39 (7.9)	10 (5.9)	4 (10.3)	4 (17.4)
5–19	138 (27.8)	40 (23.5)	7 (17.9)	5 (21.7)
20–44	155 (31.3)	55 (32.4)	15 (38.5)	6 (26.1)
45–59	86 (17.3)	30 (17.6)	7 (17.9)	3 (13.0)
≥ 60	78 (15.7)	35 (2.9)	6 (15.4)	5 (21.7)
Gender				
Female	270 (54.4)	89 (52.4)	23 (59.0)	15 (65.2)
Male	226 (45.6)	81 (47.6)	16 (41.0)	8 (34.8)

Furthermore, DENV positive samples were classified by serotypes 1–4 based on their amplified primers. Among the 170 samples positive for DENV, serotype 2 was clearly the most predominant type as it was present in 97/170 (57.1%) of samples, followed by the serotype 3 in 9/170 (5.3%). No serotypes 1 or 4 were detected (Table 2).

**Table 2:** DENV serotypes in positive samples by real time RT-PCR

Total cases of dengue n = 170 (%)	PCR real time confirmed cases				
	DENV 1 n (%)	DENV 2 n (%)	DENV 3 n (%)	DENV 4 n (%)	Undetermined n (%)
170 (34.3)	0 (0.0)	97 (57.1)	9 (5.3)	0 (0.0)	64 (37.6)

Clinical symptoms associated with the fever were registered by the attending physician in all 496 patients. Among the patients with DENV positive samples, headaches 89.4% (152/170), myalgia 86.5% (147/170) and arthralgia 84.1% (143/170) were the most common symptoms accompanying fever. In addition, sore throat and back pain were more common in patients with DENV. In the group of patients with positive samples for ZIKV the most frequent symptoms upon presentation were myalgias 89.7% (35/39), headaches 87.2% (34/39) and arthralgias 82.1% (32/39). For the patients with CHIKV, no symptoms predominance was observed; the most common symptoms were headaches, joint pain, loss of appetite, back pain, retro-ocular pain and muscle pain in 74–87% (Table 3).

**Table 3:** Clinical symptoms in patients with positive samples for DENV, ZIKV, and CHIKV

Clinical symptoms	Total n = 496 (%)	PCR real time confirmed cases		
		DENV (n = 170)	ZIKV (n = 39)	CHIKV (n = 23)
		n (%)	n (%)	n (%)
Chills	3 (0.6)	1 (0.6)	0 (0.0)	0 (0.0)
Headache	444 (89.5)	152 (89.4)	34 (87.2)	20 (87.0)
Dizziness	1 (0.2)	0 (0.0)	1 (2.6)	0 (0.0)
Cough	1 (0.2)	1 (0.6)	0 (0.0)	0 (0.0)
Sore throat	184 (37.1)	50 (29.4)	11 (28.2)	11 (47.8)
Nausea and/or vomits	251 (50.6)	86 (50.6)	20 (51.3)	13 (56.5)
Loss of appetite	312 (62.9)	104 (61.2)	23 (59.0)	18 (78.3)
Back pain	270 (54.4)	105 (61.8)	23 (59.0)	17 (73.9)
Dysuria	1 (0.2)	0 (0.0)	0 (0.0)	0 (0.0)
Myalgia	419 (84.5)	147 (86.5)	35 (89.7)	17 (73.9)
Arthralgia	396 (79.8)	143 (84.1)	32 (82.1)	19 (82.6)
Retro-ocular pain	337 (67.9)	118 (69.4)	27 (69.2)	17 (73.9)
Rash	89 (17.9)	26 (15.3)	8 (20.5)	6 (26.1)
Melena	2 (0.4)	0 (0.0)	0 (0.0)	0 (0.0)
Nasal bleeding	9 (1.8)	3 (1.8)	1 (2.6)	1 (4.3)
Gums bleeding	3 (0.6)	2 (1.2)	1 (2.6)	0 (0.0)
Petechiae	11 (2.2)	3 (1.8)	0 (0.0)	1 (4.3)
Ecchymosis	2 (0.4)	1 (0.6)	1 (2.6)	0 (0.0)
Blood tinged sputum	1 (0.2)	0 (0.0)	0 (0.0)	0 (0.0)
Abdominal pain	22 (4.4)	7 (4.1)	1 (2.6)	1 (4.3)
Thoracic pain	5 (1.0)	2 (1.2)	0 (0.0)	0 (0.0)
Fatigue	3 (0.6)	0 (0.0)	1 (2.6)	0 (0.0)
Altered mental status	1 (0.2)	1 (0.6)	0 (0.0)	1 (4.3)

### Discussion

A resurgence of arboviral diseases have been observed in Latin America and the most common etiologies are DENV, CHIKV, and ZIKV [1–3]. Peru is not an exception and despite the relatively new implementation of molecular techniques for the national epidemiological surveillance a high prevalence of these viruses have been reported in the last years with outbreaks primarily affecting the coasts of the country [7–9]. However, the low laboratory confirmation rate for the national surveillance is especially worrisome as this may lead to an underestimation of these pathogens [9, 10].

The national reports in the last 3 years have demonstrated that Piura region is an endemic area for dengue and other arboviruses [8, 9, 18]. In addition, El Niño phenomenon has produced increased rainfall and rise in temperature resulting in more mosquito breeding grounds. These climate changes have severely affected the region becoming an important scenario from arboviral diseases; thus highlighting the necessity for early detection and confirmation of febrile syndromes etiologies [23, 24]. The Peruvian epidemiological reports from July 2017 reveals that a total of 43,719 cases of dengue has been observed in the first half of the year in Piura, which represents the 64.8% of national cases. However, due to the increased demand for laboratory confirmation after El Niño phenomenon, the laboratory of the Instituto Nacional de Salud has been able to confirm only 23.2% of samples, a rate even lower than the previous year [7, 8] In addition, the most common serotypes circulating in this region are DENV-2 and DENV-3, but due to the low confirmation rates other serotypes may be neglected [7, 10].

In our study population, DENV was the most prevalent arbovirus as we were able to detect it in 34.3% of patients with acute febrile illness. Furthermore, among these 170 positive samples, we observed a clear predominance of DENV-2 in 57.1% followed by DENV-3 in 5.3%, no DENV-1 and DENV-4 were detected. This serotype distribution is similar to the national reports of 2017; however, we were not able to determine the serotype in 64 samples (37.6%) even though we used molecular characterization via real-time RT-PCR. We believe this might be explained due to possible mutations in the DENV circulating in Peru. The presence of mutant variants of DENV can occur as intra-epidemic events sometimes enhancing the replication of specific serotypes [25, 26]. This new variant of DENV may contain specific sequences which cannot be amplified by conventional primers for stereotypification [20, 26, 27].

CHIKV cases have also been increasing in 2017 after the outbreaks in Tumbes, Piura, and Ancash [7]. So far in 2017, Piura is again the most affected territory with 379 confirmed cases and 410 probable cases of CHIKV. We were able to detect CHIKV in 23 samples of patients with an acute febrile illness which represents 4.6% of our population.

Suspected autochthonous Zika cases have been reported in 10 departments of Peru and in 2017 a total of 5760 cases of ZIKV have been registered with only an 8.5% of laboratory-confirmed cases. In Piura, only 27 cases have been reported and no autochthonous cases have been confirmed [12, 28]. However, we believe that the low confirmation rate of our national surveillance may be diminishing the presence of ZIKV in North coast of Peru. In our series, we detected ZIKV in 23 of 496 samples a high number of cases if compared with the national reports in the region. However, due to the recent implementation of CHIKV and ZIKV surveillance and the lack of recent studies describing the prevalence of these viruses in Peru it is difficult for us to compare our results.

We also reported preliminary information about clinical features in patients with acute febrile illness upon presentation in our population. Among the patients with DENV positive samples, headaches 89.4% (152/170), muscle pain 86.5% (147/170) and joint pain 84.1% (143/170) were the most common symptoms accompanying fever. Similarly, for patients with ZIKV the most frequent symptoms were muscle pain 89.7% (35/39), headaches 87.2% (34/39) and joint pain 82.1% (32/39). This information demonstrates once more the similar presentation these both pathogens can have and highlights the importance of laboratory confirmation. Interestingly, for CHIKV no predominant symptom was observed being headaches, joint pain, loss of appetite, back pain, retro-ocular pain and muscle pain in 74–87% of patients. Although we observed that a sore throats and back pain were more common in patients with DENV compared to the other arboviruses, due to our limited sample we cannot conclude that these clinical features can categorically aid for a DENV diagnosis.

In conclusion, DENV is the most common arboviral cause of acute febrile illness in our study population from Piura, Peru. However, the presence of CHIKV and ZIKV should not be underestimated in the northern coast of Peru. Clinical presentations among these arboviruses can be very similar highlighting the importance of laboratory confirmation to achieve an etiological diagnosis and improve patients management. Further investigations are encouraged to improve prevalence estimates for these arboviruses and understand the causes of their resurgence. In addition, retrospective studies in stored samples should be considered to assess if Zika virus was present in Peru before 2016.

## Limitations

This study has two main limitations. First, since we only included patients from primary care facilities in an outpatient setting, it is likely that we might have neglected more severe cases. Second, since the study was designed for DENV, CHIK and ZIKV detection via real-time PCR, it is possible that other arboviral etiologies might be present in the negative samples of patients with acute febrile illness.

## Abbreviations

DENV: Dengue
CHIKV: Chikungunya
ZIKV: Zika
RT-PCR: reverse transcription polymerase chain reaction
PCR: reaccion polimerasa chain
DNA: deoxyribonucleic acid
RNA: ribonucleic acid
bp: base pairs

## References

[1] Fischer M, Staples JE, Arboviral Diseases Branch, National Center for Emerging and Zoonotic Infectious Diseases, CDC (2014). Notes from the field: Chikungunya virus spreads in the Americas—Caribbean and South America, 2013–2014. MMWR Morb Mortal Wkly Rep.

[2] Hortal M (2016). Enfermedades infecciosas emergentes y reemergentes: información actualizada. Rev Médica Urug..

[3] Forshey BM, Guevara C, Laguna-Torres VA, Cespedes M, Vargas J, Gianella A (2010). Arboviral etiologies of acute febrile illnesses in Western South America, 2000–2007. PLoS Negl Trop Dis..

[4] Mourão MP, Bastos Mde S, Figueiredo RM, Gimaque JB, Alves Vdo C, Saraiva Md (2015). Arboviral diseases in the Western Brazilian Amazon: a perspective and analysis from a tertiary health & research center in Manaus, State of Amazonas. Rev Soc Bras Med Trop.

[5] Iroh Tam PY, Obaro SK, Storch G (2016). Challenges in the etiology and diagnosis of acute febrile illness in children in low- and middle-income countries. J Pediatr Infect Dis Soc..

[6] Colombo TE, Estofolete CF, Reis AFN, da Silva NS, Aguiar ML, Cabrera EMS (2017). Clinical, laboratory and virological data from suspected ZIKV patients in an endemic arbovirus area. J Clin Virol.

[7] Ministerio de Salud, Dirección General de Epidemiología. Casos de dengue por departamento. Hasta la semana 27 del 2017. Lima: Ministerio de Salud del Perú. http://www.dge.gob.pe/portal/docs/vigilancia/boletines/2017/27.pdf. Accessed 2 July 2017.

[8] Ministerio de Salud del Peru (MINSA). Boletín Epidemiológico 03. Lima, Peru. Dirección General de Epidemiología (DGE). http://www.dge.gob.pe/portal/docs/vigilancia/boletines/2016/03.pdf. Accessed 2 Nov 2016.

[9] Ministerio de Salud del Peru (MINSA) Casos de Dengue por departamentos Peru 2017. Lima, Peru. Dirección General de Epidemiología (DGE). http://www.dge.gob.pe/portal/docs/vigilancia/sala/2017/SE02/dengue.pdf. Accessed 28 Aug 2017.

[10] Red Nacional de Epidemiologia (RENACE) Vigilancia del síndrome febril en áreas de alto riesgo de transmisión de enfermedades infeccionsas de impacto en salud publica en el Peru. Lima, Perú. Oficina General de Epidemiología (OGE). http://www.dge.gob.pe/publicaciones/pub_invepi/iepi05.pdf. Accessed 09 Nov 2016.

[11] Nunes MRT, Faria NR, de Vasconcelos JM, Golding N, Kraemer MU, de Oliveira LF (2015). Emergence and potential for spread of Chikungunya virus in Brazil. BMC Med..

[12] Organization WH, et al. PAHO. 2015. Number of reported cases of Chikungunya Fever in the Americas—EW 35. http://www.paho.org/hq/index.php?option=com_topics&view=readall&cid=5927&Itemid=40931&lang=en. Accessed 4 Sept 2015.

[13] Ministerio de Salud, Dirección General de Epidemiología. Primer caso autóctono de Chikungunya y Riesgo de Transmisión en el Perú Lima: Ministerio de Salud del Perú. http://www.dge.gob.pe/portal/docs/alertas/2015/AE005.pdf. Accessed 11 June 2015.

[14] Instituto Nacional de Defensa Civil (INDECI) Reporte de Información del Ministerio de Salud como consecuencia del Niño Costero [Internet]. Lima, Peru. Ministerio de Salud (MINSA). http://www.indeci.gob.pe/objetos/noticias/NTY=/NTE1Mw==/fil20170512180616.pdf. Accessed 28 Aug 2017.

[15] Ministerio de Salud, Dirección General de Epidemiología. Boletín epidemiológico del Perú SE 07. http://www.dge.gob.pe/portal/docs/vigilancia/boletines/2017/07.pdf. Accessed 11 Feb 2017.

[16] Pan American Health Organization/World Health Organization. Zika—Epidemiological Report Peru. June 2017. Washington, D.C.: PAHO/WHO. http://www.paho.org/hq/index.php?option=com_docman&task=doc_view&gid=35138&Itemid=270. Accessed June 2017.

[17] Thiboutot MM, Kannan S, Kawalekar OU, Shedlock DJ, Khan AS, Sarangan G (2010). Chikungunya: a potentially emerging epidemic?. PLoS Negl Trop Dis..

[18] Afreen N, Deeba F, Khan WH, Haider SH, Kazim SN, Ishrat R (2014). Molecular characterization of dengue and Chikungunya virus strains circulating in New Delhi, India. Microbiol Immunol.

[19] Red Nacional de Epidemiologia (RENACE). Casos de Dengue por Departamentos Peru 2016. Lima, Perú. Dirección General de Epidemiología (DGE). http://www.dge.gob.pe/portal/docs/vigilancia/sala/2016/SE01/dengue.pdf. Accessed 29 Oct 2016.

[20] Leparc-Goffart I, Baragatti M, Temmam S (2009). Development and validation of real-time one-step reverse transcription-PCR for the detection and typing of dengue viruses. J Clin Virol.

[21] Peyrefitte CN, Pastorino BA, Bessaud M, Gravier P (2005). Dengue type 3 virus, Saint Martin, 2003–2004. Emerg Infect Dis.

[22] Faye O, Faye O, Diallo D (2013). Quantitative real-time PCR detection of Zika virus and evaluation with field-caught mosquitoes. Virol J..

[23] Tantaléan-Yépez D, Sánchez-Carbonel J, Ulloa-Urizar G, Aguilar-Luis MA, Espinoza-Morales D, Silva-Caso W (2016). Arboviruses emerging in Peru: need for early detection of febrile syndrome during El Niño episodes. Asian Pac J Trop Med..

[24] Cabezas C, Fiestas V, García-Mendoza M, Palomino M, Mamani E, Donaires F (2015). Dengue in Peru: a quarter century after its reemergence. Rev Peru Med Exp Salud Publica..

[25] Blaney J, Manipon G, Firestone C, Johnson D, Hanson C, Murphy B (2003). Mutations which enhance the replication of dengue virus type 4 and an antigenic chimeric dengue virus type 2/4 vaccine candidate in Vero cells. Vaccine..

[26] Hapuarachchi H, Koo C, Kek R, Xu H, Lai Y, Liu L (2016). Intra-epidemic evolutionary dynamics of a Dengue virus type 1 population reveal mutant spectra that correlate with disease transmission. Sci Rep..

[27] Sessions O, Wilm A, Kamaraj U, Choy M, Chow A, Chong Y (2015). Analysis of dengue virus genetic diversity during human and mosquito infection reveals genetic constraints. PLoS Negl Trop Dis..

[28] Ministerio de Salud del Peru (MINSA) Casos de Zika Perú 2017. Lima, Peru. Dirección General de Epidemiología (DGE). http://www.dge.gob.pe/portal/docs/vigilancia/sala/2017/SE23/zika.pdf. Accessed 29 Aug 2017.

